# 4-Oxo-*N*-phenyl-1,4-di­hydro­pyridine-3-carboxamide

**DOI:** 10.1107/S2414314623006016

**Published:** 2023-07-14

**Authors:** Xi Shu, Sihui Long

**Affiliations:** a School of Chemical Engineering and Pharmacy, Wuhan Institute of Technology, Wuhan, Hubei 430205, People’s Republic of China; University Koblenz-Landau, Germany

**Keywords:** crystal structure, hydrogen bonding

## Abstract

The title compound, C_12_H_10_N_2_O_2_, shows a nearly planar conformation. The crystal structure is sustained by hydrogen bonds between the NH and the carbonyl O function of the 4-oxo-1,4-di­hydro­pyridine ring of the mol­ecules, forming infinite chains along the *b-*axis direction.

## Abstract 2

Single crystals of 4-oxo-*N*-phenyl-1,4-di­hydro­pyridine-3-carboxamide were obtained by slow evaporation of a solution of the title compound in acetone. The crystal structure is sustained by hydrogen bonds between the NH and the carbonyl O function of the 4-oxo-1,4-di­hydro­pyridine ring of the mol­ecules, forming infinite chains along the *b-*axis direction.

## Graphical abstract

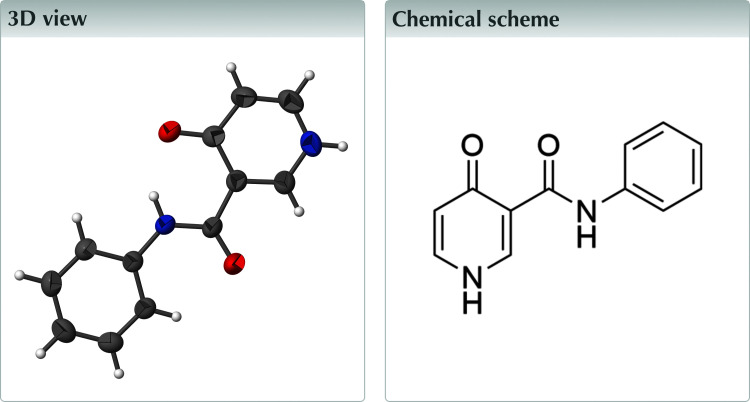

## Structure description

**Figure 1:**
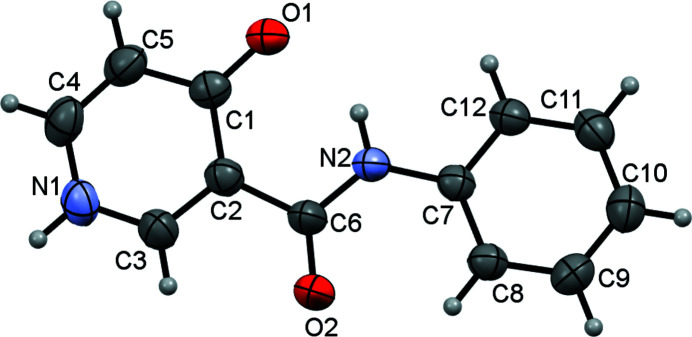
Mol­ecular structure of the title compound, with displacement ellipsoids drawn at the 50% probability level.

**Figure 2:**
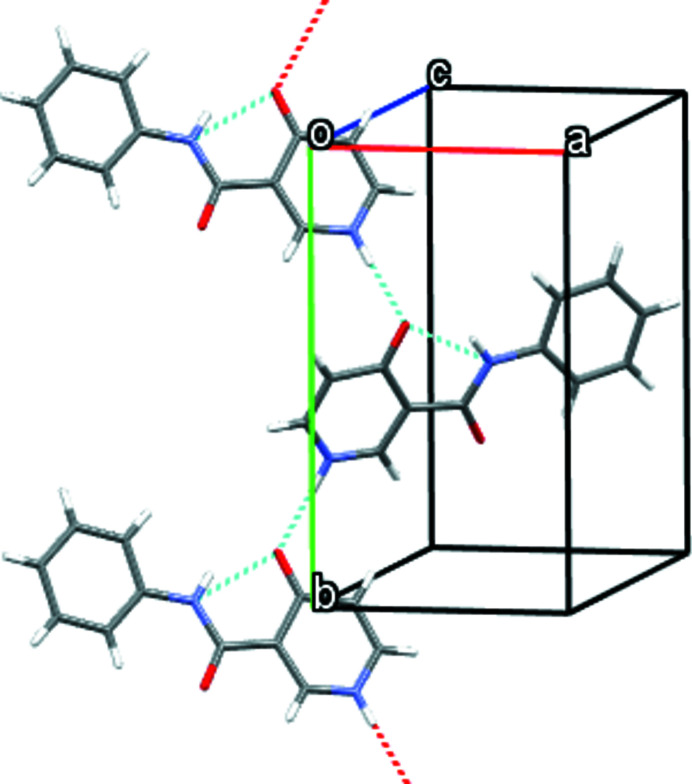
Packing of the mol­ecules in the title compound viewed along the *b* axis.

The title compound (Fig. 1 ▸) is a derivative of *N*-phenyl­nicotinamide, which is an efficient molluscicide (Dunlop *et al.*, 1980 ▸). In addition, the compound can be used as a raw material for many chemical reactions. We were inter­ested in its solid-state behavior since it is a structural isomer of *N*-phenyl-2-hy­droxy­nicotinanilide, which has inter­esting structural properties (Zhoujin *et al.*, 2021 ▸). In our study, the compound was synthesized by an amide condensation reaction (Narajan *et al.*, 2016 ▸), and single crystals were obtained by slow evaporation of an acetone solution of the compound. The compound has a nearly planar conformation as evidenced by the dihedral angle between the 4-oxo-1,4-di­hydro­pyridine and benzene rings of 6.80 (8)°. An intra­molecular hydrogen bond is formed between the NH of the amide and the carbonyl O atom on the 4-oxo-1,4-di­hydro­pyridine ring. In the crystal, the mol­ecules form chains along the *b-*axis direction through hydrogen bonds between the NH group and the carbonyl O atom of the 4-oxo-1,4-di­hydro­pyridine ring (Fig. 2 ▸, Table 1 ▸).

## Synthesis and crystallization

**Figure 3:**
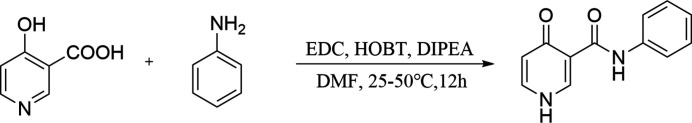
Reaction scheme.

**Figure 4:**
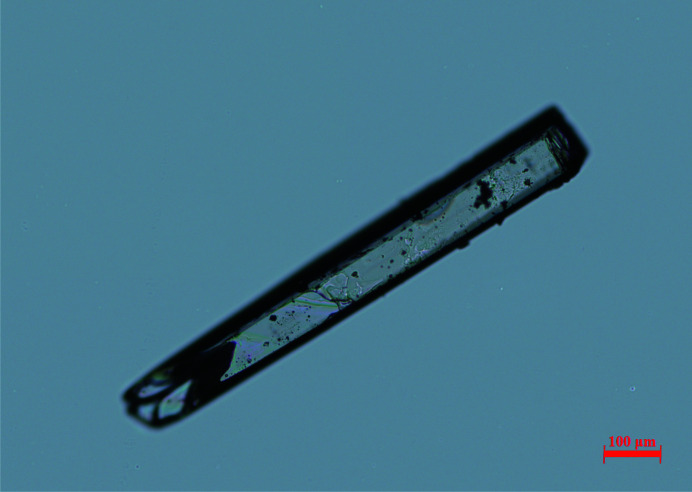
A representative crystal of the title compound.

4-Hy­droxy­nicotinic acid (0.51 g, 3.67 mmol), 1-ethyl-3-(3-di­methyl­amino­prop­yl)carbo­di­imide hydro­chloride (EDC, 1.06 g, 5.51 mmol) and hy­droxy­benzotriazole (HOBT, 0.60 g, 4.40 mmol) were dissolved in 6 ml of DMF and stirred at 0°C for 1 h. Then diiso­propyl­ethyl­amine (DIPEA, 0.95 g, 7.34 mmol) and aniline (0.28 ml, 3.67 mmol) were added and the reaction was completed under continuous stirring at 50°C for 12 h. Then 20 ml of deionized water were added to the reaction mixture, which was placed into a refrigerator at 5°C overnight. The resulting precipitate was collected by filtration and washed with deionized water to obtain 0.306 g (39% based on 4-hy­droxy­nicotinic acid) of the title compound (Fig. 3 ▸). The obtained compound was fully dissolved in acetone under ultrasound until a clear solution was obtained, which was then filtered into a transparent glass bottle. The bottle was placed in a fume hood for slow evaporation of the solvent. Colorless rod-shaped crystals (Fig. 4 ▸) were obtained in a few days.

## Refinement

Crystal data, data collection and structure refinement details are summarized in Table 2 ▸.

## Supplementary Material

Crystal structure: contains datablock(s) global, I. DOI: 10.1107/S2414314623006016/im4020sup1.cif

Structure factors: contains datablock(s) I. DOI: 10.1107/S2414314623006016/im4020Isup2.hkl

Supporting information file. DOI: 10.1107/S2414314623006016/im4020Isup3.cml

CCDC reference: 2280191

Additional supporting information:  crystallographic information; 3D view; checkCIF report

## Figures and Tables

**Table 1:** Hydrogen-bond geometry (Å, °)

*D*—H⋯*A*	*D*—H	H⋯*A*	*D*⋯*A*	*D*—H⋯*A*
N1—H1⋯O1^i^	0.91 (2)	1.84 (3)	2.744 (2)	171 (2)
C8—H8⋯O2	0.93	2.27	2.861 (2)	121
N2—H2⋯O1	0.88 (3)	1.91 (2)	2.6731 (18)	144 (2)

Symmetry code: (i) 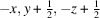 .

**Table 2:** Experimental details

Crystal data	
Chemical formula	C_12_H_10_N_2_O_2_
*M*_r_	214.22
Crystal system, space group	Orthorhombic, *P*2_1_2_1_2_1_
Temperature (K)	276
*a*, *b*, *c* (Å)	6.68228 (10), 11.79159 (18), 13.1717 (2)
*V* (Å^3^)	1037.86 (3)
*Z*	4
Radiation type	Cu *K*α
μ (mm^−1^)	0.79
Crystal size (mm)	0.1 × 0.07 × 0.05

Data collection	
Diffractometer	Rigaku Oxford Diffraction, Synergy Custom system, HyPix
Absorption correction	Multi-scan (*CrysAlis PRO*; Rigaku OD, 2021 ▸)
*T*_min_, *T*_max_	0.864, 1.000
No. of measured, independent and observed [*I* > 2σ(*I*)] reflections	6101, 2075, 2003
*R*_int_	0.019
(sin θ/λ)_max_ (Å^−1^)	0.632

Refinement	
*R*[*F* ^2^ > 2σ(*F* ^2^)], *wR*(*F* ^2^), *S*	0.030, 0.083, 1.06
No. of reflections	2075
No. of parameters	154
H-atom treatment	H atoms treated by a mixture of independent and constrained refinement
Δρ_max_, Δρ_min_ (e Å^−3^)	0.21, −0.14
Absolute structure	Flack *x* determined using 788 quotients [(*I* ^+^)−(*I* ^−^)]/[(*I* ^+^)+(*I* ^−^)] (Parsons *et al.*, 2013 ▸)
Absolute structure parameter	0.11 (8)

Computer programs: *CrysAlis PRO* (Rigaku OD, 2021 ▸), *SHELXT* (Sheldrick, 2015*a* ▸), *SHELXL2018/3* (Sheldrick, 2015*b* ▸), *OLEX2* (Dolomanov *et al.*, 2009 ▸) and *Mercury* (Macrae *et al.*, 2020 ▸).

## full crystallographic data

### Crystal data

**Table:**

C_12_H_10_N_2_O_2_	*D*_x_ = 1.371 Mg m^−^^3^
*M**_r_* = 214.22	Cu *K*α radiation, λ = 1.54184 Å
Orthorhombic, *P*2_1_2_1_2_1_	Cell parameters from 5537 reflections
*a* = 6.68228 (10) Å	θ = 3.3–77.1°
*b* = 11.79159 (18) Å	µ = 0.79 mm^−^^1^
*c* = 13.1717 (2) Å	*T* = 276 K
*V* = 1037.86 (3) Å^3^	Block, clear light colourless
*Z* = 4	0.1 × 0.07 × 0.05 mm
*F*(000) = 448	

### Data collection

**Table:**

Rigaku Oxford Diffraction, Synergy Custom system, HyPix diffractometer	2075 independent reflections
Radiation source: Rotating-anode X-ray tube, Rigaku (Cu) X-ray Source	2003 reflections with *I* > 2σ(*I*)
Mirror monochromator	*R*_int_ = 0.019
Detector resolution: 10.0000 pixels mm^-1^	θ_max_ = 77.2°, θ_min_ = 5.0°
ω scans	*h* = −8→8
Absorption correction: multi-scan (CrysAlisPro; Rigaku OD, 2021)	*k* = −13→14
*T*_min_ = 0.864, *T*_max_ = 1.000	*l* = −14→16
6101 measured reflections	

### Refinement

**Table:**

Refinement on *F*^2^	H atoms treated by a mixture of independent and constrained refinement
Least-squares matrix: full	*w* = 1/[σ^2^(*F*_o_^2^) + (0.0478*P*)^2^ + 0.0929*P*] where *P* = (*F*_o_^2^ + 2*F*_c_^2^)/3
*R*[*F*^2^ > 2σ(*F*^2^)] = 0.030	(Δ/σ)_max_ < 0.001
*wR*(*F*^2^) = 0.083	Δρ_max_ = 0.21 e Å^−^^3^
*S* = 1.06	Δρ_min_ = −0.14 e Å^−^^3^
2075 reflections	Extinction correction: SHELXL-2018/3 (Sheldrick 2015b), Fc^*^=kFc[1+0.001xFc^2^λ^3^/sin(2θ)]^-1/4^
154 parameters	Extinction coefficient: 0.027 (3)
0 restraints	Absolute structure: Flack *x* determined using 788 quotients [(*I*^+^)-(*I*^-^)]/[(*I*^+^)+(*I*^-^)] (Parsons *et al.*, 2013)
Primary atom site location: dual	Absolute structure parameter: 0.11 (8)
Hydrogen site location: mixed	

### Special details

**Table:**

Geometry. All esds (except the esd in the dihedral angle between two l.s. planes) are estimated using the full covariance matrix. The cell esds are taken into account individually in the estimation of esds in distances, angles and torsion angles; correlations between esds in cell parameters are only used when they are defined by crystal symmetry. An approximate (isotropic) treatment of cell esds is used for estimating esds involving l.s. planes.
Refinement. The positions of H atoms at N1 and N2 were obtained from the difference Fourier map and were refined freely. Other H atoms were positioned geometrically with C—H = 0.93 Å and constrained to ride on their parent atoms, with *U*_iso_(H) = 1.2 *U*_eq_(C) (Table 2).

### Fractional atomic coordinates and isotropic or equivalent isotropic displacement parameters (Å^2^)

**Table:**

	*x*	*y*	*z*	*U*_iso_*/*U*_eq_	
O1	0.25139 (19)	0.41568 (10)	0.25242 (11)	0.0504 (4)	
O2	0.4568 (3)	0.68655 (14)	0.4334 (2)	0.0985 (8)	
N1	−0.0559 (2)	0.71242 (14)	0.26837 (14)	0.0522 (4)	
H1	−0.122 (4)	0.780 (2)	0.2688 (18)	0.060 (6)*	
N2	0.5233 (2)	0.50907 (12)	0.37556 (12)	0.0418 (3)	
C1	0.1596 (3)	0.50951 (14)	0.25749 (13)	0.0415 (4)	
C2	0.2281 (3)	0.60428 (14)	0.31705 (14)	0.0417 (4)	
C3	0.1157 (3)	0.70093 (15)	0.32024 (16)	0.0497 (4)	
H3	0.159842	0.761165	0.359932	0.060*	
C4	−0.1229 (3)	0.62714 (18)	0.20986 (16)	0.0553 (5)	
H4	−0.240686	0.636483	0.173182	0.066*	
C5	−0.0226 (3)	0.52847 (17)	0.20341 (15)	0.0534 (5)	
H5	−0.073310	0.470945	0.162547	0.064*	
C6	0.4136 (3)	0.60500 (15)	0.38116 (17)	0.0485 (5)	
C7	0.7012 (2)	0.48310 (14)	0.42740 (12)	0.0372 (4)	
C8	0.8102 (3)	0.56149 (14)	0.48339 (13)	0.0414 (4)	
H8	0.766564	0.636194	0.488177	0.050*	
C9	0.9846 (3)	0.52788 (17)	0.53218 (15)	0.0489 (4)	
H9	1.056878	0.580418	0.569958	0.059*	
C10	1.0519 (3)	0.41749 (18)	0.52534 (16)	0.0534 (5)	
H10	1.168367	0.395585	0.558657	0.064*	
C11	0.9447 (3)	0.33961 (16)	0.46848 (15)	0.0512 (5)	
H11	0.989888	0.265248	0.463118	0.061*	
C12	0.7708 (3)	0.37210 (15)	0.41965 (14)	0.0447 (4)	
H12	0.699818	0.319469	0.381348	0.054*	
H2	0.471 (3)	0.455 (2)	0.3385 (19)	0.061 (7)*	

### Atomic displacement parameters (Å^2^)

**Table:**

	*U*^11^	*U*^22^	*U*^33^	*U*^12^	*U*^13^	*U*^23^
O1	0.0506 (7)	0.0403 (6)	0.0602 (8)	−0.0014 (5)	−0.0053 (6)	−0.0110 (5)
O2	0.0752 (11)	0.0582 (10)	0.162 (2)	0.0273 (8)	−0.0592 (12)	−0.0567 (11)
N1	0.0498 (8)	0.0454 (8)	0.0614 (10)	0.0053 (7)	−0.0058 (7)	0.0124 (7)
N2	0.0383 (7)	0.0354 (7)	0.0518 (8)	−0.0002 (6)	−0.0030 (6)	−0.0095 (6)
C1	0.0434 (8)	0.0399 (8)	0.0411 (8)	−0.0060 (7)	0.0023 (7)	0.0022 (7)
C2	0.0404 (8)	0.0369 (8)	0.0478 (9)	−0.0013 (6)	0.0012 (7)	0.0006 (7)
C3	0.0482 (9)	0.0395 (9)	0.0613 (11)	0.0018 (7)	−0.0056 (8)	0.0019 (8)
C4	0.0516 (10)	0.0588 (11)	0.0555 (11)	−0.0042 (9)	−0.0138 (9)	0.0172 (9)
C5	0.0586 (11)	0.0510 (10)	0.0506 (10)	−0.0091 (9)	−0.0146 (9)	0.0035 (8)
C6	0.0429 (9)	0.0349 (8)	0.0677 (12)	0.0013 (7)	−0.0071 (8)	−0.0117 (8)
C7	0.0363 (7)	0.0371 (8)	0.0383 (7)	−0.0004 (6)	0.0048 (6)	−0.0016 (6)
C8	0.0422 (8)	0.0374 (8)	0.0445 (9)	−0.0024 (6)	0.0020 (7)	−0.0028 (7)
C9	0.0469 (9)	0.0532 (10)	0.0465 (9)	−0.0069 (8)	−0.0044 (8)	−0.0033 (8)
C10	0.0470 (9)	0.0621 (11)	0.0510 (10)	0.0061 (8)	−0.0060 (8)	0.0042 (9)
C11	0.0550 (10)	0.0451 (9)	0.0536 (11)	0.0116 (8)	0.0016 (9)	−0.0004 (8)
C12	0.0483 (9)	0.0376 (8)	0.0482 (9)	0.0002 (7)	−0.0003 (8)	−0.0051 (7)

### Geometric parameters (Å, º)

**Table:**

O1—C1	1.267 (2)	C4—C5	1.346 (3)
O2—C6	1.217 (2)	C5—H5	0.9300
N1—H1	0.91 (2)	C7—C8	1.389 (2)
N1—C3	1.341 (3)	C7—C12	1.393 (2)
N1—C4	1.344 (3)	C8—H8	0.9300
N2—C6	1.350 (2)	C8—C9	1.388 (2)
N2—C7	1.405 (2)	C9—H9	0.9300
N2—H2	0.88 (3)	C9—C10	1.380 (3)
C1—C2	1.440 (2)	C10—H10	0.9300
C1—C5	1.428 (2)	C10—C11	1.384 (3)
C2—C3	1.365 (2)	C11—H11	0.9300
C2—C6	1.500 (2)	C11—C12	1.382 (3)
C3—H3	0.9300	C12—H12	0.9300
C4—H4	0.9300		
			
C3—N1—H1	120.0 (15)	O2—C6—N2	124.36 (17)
C3—N1—C4	120.09 (17)	O2—C6—C2	121.23 (16)
C4—N1—H1	119.8 (15)	N2—C6—C2	114.41 (15)
C6—N2—C7	128.00 (15)	C8—C7—N2	123.83 (15)
C6—N2—H2	115.0 (16)	C8—C7—C12	119.29 (15)
C7—N2—H2	116.8 (16)	C12—C7—N2	116.87 (15)
O1—C1—C2	123.54 (16)	C7—C8—H8	120.1
O1—C1—C5	121.55 (16)	C9—C8—C7	119.73 (16)
C5—C1—C2	114.91 (16)	C9—C8—H8	120.1
C1—C2—C6	125.01 (15)	C8—C9—H9	119.6
C3—C2—C1	119.31 (16)	C10—C9—C8	120.84 (17)
C3—C2—C6	115.63 (15)	C10—C9—H9	119.6
N1—C3—C2	122.60 (17)	C9—C10—H10	120.3
N1—C3—H3	118.7	C9—C10—C11	119.48 (17)
C2—C3—H3	118.7	C11—C10—H10	120.3
N1—C4—H4	119.4	C10—C11—H11	119.9
N1—C4—C5	121.13 (17)	C12—C11—C10	120.18 (18)
C5—C4—H4	119.4	C12—C11—H11	119.9
C1—C5—H5	119.0	C7—C12—H12	119.8
C4—C5—C1	121.92 (17)	C11—C12—C7	120.47 (17)
C4—C5—H5	119.0	C11—C12—H12	119.8
			
O1—C1—C2—C3	−178.02 (16)	C5—C1—C2—C3	1.9 (3)
O1—C1—C2—C6	−0.6 (3)	C5—C1—C2—C6	179.37 (17)
O1—C1—C5—C4	178.76 (18)	C6—N2—C7—C8	−9.9 (3)
N1—C4—C5—C1	−0.4 (3)	C6—N2—C7—C12	171.15 (18)
N2—C7—C8—C9	179.95 (16)	C6—C2—C3—N1	−178.90 (18)
N2—C7—C12—C11	−179.93 (16)	C7—N2—C6—O2	0.9 (4)
C1—C2—C3—N1	−1.2 (3)	C7—N2—C6—C2	−178.75 (16)
C1—C2—C6—O2	−176.4 (2)	C7—C8—C9—C10	0.4 (3)
C1—C2—C6—N2	3.3 (3)	C8—C7—C12—C11	1.1 (3)
C2—C1—C5—C4	−1.2 (3)	C8—C9—C10—C11	0.4 (3)
C3—N1—C4—C5	1.2 (3)	C9—C10—C11—C12	−0.5 (3)
C3—C2—C6—O2	1.2 (3)	C10—C11—C12—C7	−0.3 (3)
C3—C2—C6—N2	−179.15 (17)	C12—C7—C8—C9	−1.1 (2)
C4—N1—C3—C2	−0.4 (3)		

### Hydrogen-bond geometry (Å, º)

**Table:**

*D*—H···*A*	*D*—H	H···*A*	*D*···*A*	*D*—H···*A*
N1—H1···O1^i^	0.91 (2)	1.84 (3)	2.744 (2)	171 (2)
C8—H8···O2	0.93	2.27	2.861 (2)	121
N2—H2···O1	0.88 (3)	1.91 (2)	2.6731 (18)	144 (2)

Symmetry code: (i) −*x*, *y*+1/2, −*z*+1/2.
